# STRIDER: Sildenafil therapy in dismal prognosis early-onset intrauterine growth restriction – a protocol for a systematic review with individual participant data and aggregate data meta-analysis and trial sequential analysis

**DOI:** 10.1186/2046-4053-3-23

**Published:** 2014-03-11

**Authors:** Wessel Ganzevoort, Zarko Alfirevic, Peter von Dadelszen, Louise Kenny, Aris Papageorghiou, Aleid van Wassenaer-Leemhuis, Christian Gluud, Ben Willem Mol, Philip N Baker

**Affiliations:** 1 Department of Obstetrics and Gynecology, Academic Medical Centre, Room: H4-278, PO BOX 22660, 1100 DD Amsterdam, The Netherlands; 2 Department of Women’s and Children’s Health, Liverpool Women’s Hospital, University of Liverpool, Crown Street, Liverpool L8 7SS, UK; 3 Department of Obstetrics and Gynaecology, University of British Colombia, Rm V3-339, 950 West 28th Avenue, Vancouver, BC V5Z 4H4, Canada; 4 The Irish Centre for Fetal and Neonatal Translational Research, University College Cork, Cork, Ireland; 5 Department of Obstetrics and Fetal Medicine, St George’s Hospital, Blackshaw Road, London SW17 0QT, UK; 6 Department of Neonatology, Academic Medical Centre, PO BOX 22660, 1100 DD Amsterdam, The Netherlands; 7 Department 7812, Copenhagen Trial Unit, Centre for Clinical Intervention Research, Rigshospitalet, Copenhagen University Hospital, Blegdamsvej 9, DK 2100 Copenhagen, Denmark; 8 Department of Obstetrics and Gynaecology, Academic Medical Centre, Room H4-213, PO Box 22700, 1105 DE Amsterdam, The Netherlands; 9 Liggins Institute, University of Auckland, Level 2, Building 505, 85 Park Rd, Grafton, Private Bag 92019, Auckland 1142, New Zealand

**Keywords:** Adverse neonatal outcome, Fetal growth restriction, Individual participant data meta-analysis, Sildenafil

## Abstract

**Background:**

In pregnancies complicated by early-onset extreme fetal growth restriction, there is a high risk of preterm birth and an overall dismal fetal prognosis. Sildenafil has been suggested to improve this prognosis. The first aim of this review is to assess whether sildenafil benefits or harms these babies. The second aim is to analyse if these effects are modified in a clinically meaningful way by factors related to the women or the trial protocol.

**Methods/Design:**

The STRIDER (Sildenafil Therapy In Dismal prognosis Early-onset intrauterine growth Restriction) Individual Participant Data (IPD) Study Group will conduct a prospective IPD and aggregate data systematic review with meta-analysis and trial sequential analysis. The STRIDER IPD Study Group started trial planning and funding applications in 2012. Three trials will be launched in 2014, recruiting for three years. Further trials are planned to commence in 2015.

The primary outcome for babies is being alive at term gestation without evidence of serious adverse neonatal outcome. The latter is defined as severe central nervous system injury (severe intraventricular haemorrhage (grade 3 and 4) or cystic periventricular leukomalacia, demonstrated by ultrasound and/or magnetic resonance imaging) or other severe morbidity (bronchopulmonary dysplasia, retinopathy of prematurity requiring treatment, or necrotising enterocolitis requiring surgery). The secondary outcomes are improved fetal growth velocity assessed by ultrasound abdominal circumference measurements, gestational age and birth weight (centile) at delivery, and age-adequate performance on the two-year Bayley scales of infant and toddler development-III (composite cognitive score and composite motor score). Subgroup and sensitivity analyses in the IPD meta-analysis include assessment of the influence of several patient characteristics: an abnormal or normal serum level of placental growth factor, absent/reversed umbilical arterial end diastolic flow at commencement of treatment, and other patient characteristics available at baseline such as gestational age and estimated fetal weight. The secondary outcomes for mothers include co-incidence and severity of the maternal syndrome of pre-eclampsia, mortality, and other serious adverse events.

**Discussion:**

Trials are expected to start in 2013–2014 and end in 2016–2017. Data analyses of individual trials are expected to finish in 2019. Given the pre-planned and agreed IPD protocol, these results should be available in 2020.

## Background

Severe early-onset fetal growth restriction (FGR) complicates approximately 0.4% of pregnancies and severely increases the risk of perinatal morbidity and mortality. This is particularly due to premature delivery, both for fetal and for secondary maternal indications such as the development of pre-eclampsia [1]. Placental disease, consequent on deficient uteroplacental blood flow, includes FGR, pre-eclampsia, and placental abruption and has been implicated in more than 50% of iatrogenic premature births [2]. For this reason, the problem of severe FGR forms a substantial portion of the population that tertiary care centres care for.

The effect of severe early-onset FGR is particularly significant: of those born alive, less than a third will survive their neonatal intensive care unit (NICU) stay without significant neurodevelopmental sequelae. Survival rates for severely growth-restricted fetuses very remote from term (<28 weeks’ gestation) vary from 7% to 33% [1,3,4].

As these early-onset FGR children are born very preterm, there are significant risks of neonatal mortality, major and minor morbidity, and long-term health sequelae [5,6]. These are not only strongly gestational-age related [7], but are also related to FGR [8]. Although most of the surviving children are without severe disabilities at school age, many of them meet serious difficulties in everyday life and the burden of mild developmental abnormalities and behavioural and learning disorders increases with age. In adolescents, as many as 40% of the survivors will not be able to become fully independent adults [9].

The direct costs of care include the increased cost of intensive antenatal surveillance (with or without a period of hospitalisation), caesarean delivery (if considered viable), NICU care, routine post-NICU follow-up, need for special schooling and educational support (speech therapy, physical therapy, psychological support), and, often, specialised neurodevelopmental assessments and interventions. Such costs are in addition to the indirect costs to families (grief, lost wages, supportive services, and relationship stress) and represent a considerable economic burden. Safe pregnancy prolongation accompanied by additional fetal growth would be associated with significant societal and financial savings.

The use of ultrasound Doppler waveform analysis in pregnancies complicated by FGR suggests compromised uteroplacental circulation and placental hypoperfusion. Currently there are no specific evidence-based therapies for placental insufficiency and early-onset severe FGR. Non-specific interventions include primarily lifestyle modifications, such as reducing or stopping work, stopping aerobic exercise, rest at home, and hospital admission for rest and surveillance. These interventions, which are not supported by evidence from randomised trials, are used in the belief that rest will enhance the uteroplacental circulation at the expense of that to the glutei and quadriceps muscles.

There is evidence from *ex vivo* and animal models of growth restriction that the phosphodiesterase 5 inhibitor sildenafil citrate increases average pup birth weight and improves uteroplacental blood flow (umbilical artery, uterine artery) [10-12]. Sildenafil citrate may thus offer a potential therapeutic strategy to improve uteroplacental blood flow in human pregnancies complicated by severe FGR [12]. There is evidence, albeit limited, to support the use of sildenafil in human pregnancy. A small trial in women with early-onset pre-eclampsia (a maternal presentation of placental compromise) showed no resolution of pre-eclampsia, but an effect on birth weight [13]. In addition, a small cohort study showed a tendency towards more live-born children that survived intact to primary discharge after sildenafil intervention [14]. From all observations thus far, there are no indications that sildenafil in the second trimester has significant fetotoxicity.

Coordinated initiatives have been launched and approved/shortlisted for funding in networks in the United Kingdom/Ireland, The Netherlands, and New Zealand/Australia. Initiatives in Canada and the United States of America are in an earlier phase. These trials are embedded in a global network (Global Obstetrics Network – GONet) [15]. This network was initiated to provide a forum for international interaction and foster communication between groups that perform clinical studies in obstetrics. GONet has started the collaborative initiative to resolve the underlying question: does sildenafil versus placebo improve outcomes in early-onset severely growth restricted fetuses with a dismal prognosis? Within each part of the network, stand-alone randomised clinical trials will be conducted; a prospective systematic review with meta-analyses and trial sequential analyses of individual participant data (IPD) and aggregate data will then be performed. The concerted effort in the design and conduct of these large international studies provides an efficient use of scarce resources and will optimise resolution of the research questions, in addition to facilitating adequate power for subgroup analyses.

## Methods/Design

### Study design

Prospectively planned systematic review with IPD and aggregate data meta-analysis [16] and trial sequential analysis [17-21] of randomised clinical trials comparing sildenafil with matching placebo tablets or no intervention for treatment of severe early-onset FGR.

### Inclusion and exclusion criteria for the studies

Studies, published or unpublished, will be included if they are randomised clinical trials. Other study designs for assessment of benefits will not be included. For the assessment of harm we will also include observational studies. Given the pre-planned nature of the IPD meta-analysis, most or all studies will have been identified before the start of the individual trials and will participate in central data collection. If other trials, yet unknown, are conducted simultaneously, their eligibility will be assessed independently and unblinded for author and journal by two members of the Sildenafil Therapy In Dismal prognosis Early-onset intrauterine growth Restriction (STRIDER) IPD Study Group. Any differences in opinion regarding eligibility will be resolved by discussion. If the study design is suboptimal for IPD meta-analysis or the authors do not cooperate, the study will be included for aggregate data analysis.

We will use the Cochrane Central Register of Controlled Trials (CENTRAL), MEDLINE, Embase, LILACS, and Science Citation Index Expanded using the terms “fetal growth retardation” and “sildenafil”. In addition, we will access the WHO Trial Portal [22], Current Controlled Trials [23], the Australian and New Zealand Trials Register [24], and the ISRCTN register [25] to identify recently completed or ongoing trials. Experts in the field and trialists will be asked if they know of any unpublished or other trials. The currently known trials that will be conducted and included are:

*UK/Ireland:* Planned sample size 112; primary outcome: prolongation of gestational age at birth.

*The Netherlands:* Planned sample size 354; primary outcome: infant survival to term age without evidence of serious adverse neonatal outcome.

*New Zealand/Australia:* Planned sample size 122; primary outcome: fetal growth velocity.

*Canada:* Planned sample size 90; primary outcome: fetal growth velocity.

### Types of participants

Women with a singleton pregnancy between 20 + 0 and 29 + 6 weeks with severe fetal growth restriction of likely placental origin, and with estimated significant likelihood of fetal/neonatal death.

### Type of intervention

Sildenafil versus placebo tablet orally or versus no intervention until fetal death, delivery, or 32 weeks of gestation (whichever comes first). Co-interventions will be allowed provided they are administered similarly in the intervention groups.

### Data collection and management

The investigators of each individual trial have designed each trial together before launch and patient recruitment. All of the listed eligible trials that have agreed to join the STRIDER Study Group have agreed a common core data set and have agreed to supply their trial’s IPD to a central repository. Randomisation will be centrally controlled using an on-line computerised randomisation service. All have agreed to collect all relevant variables on baseline characteristics and outcomes (the primary and secondary outcomes of this protocol). All have agreed to centrally collect data. The University of British Columbia (UBC) will host data management through the UBC Perinatal Clinical Trials Unit. Wherever possible, data will be collected as continuous rather than dichotomous measurements. All trialists have agreed to collect information on the number of eligible but not-included patients. If the patient consents, a number of relevant baseline and outcome parameters will be collected.

If other relevant randomised clinical trials are identified, data collected from those studies will include all women randomised and coded for anonymity (date of birth, centre identification), baseline data for descriptive purposes and analyses, details of the intervention given (date of randomisation, allocated intervention), and outcomes, to allow planned analyses. Trialists will provide non-identified IPD in any convenient format by encrypted, electronic transfer where possible or other means as needed. The individual trial data will be recoded as required, and stored in the secure database that will only be accessible by authorised personnel of the UBC Perinatal Clinical Trials Unit. Trialists will be asked to verify all recoded data prior to any analysis and the data will not be used for any other purpose without permission of all collaborators.

The data will be checked with respect to range, internal consistency, missing or extreme values, errors, and consistency with published reports. Trial details, such as randomisation methods, blinding, and intervention details, will be crosschecked against published reports, trial protocols, and data collection sheets. Inconsistencies or missing data will be discussed with the individual trialists and attempts will be made to resolve any problems by consensus.

### Data items to be collected

#### Trial level information

1. Dates the trial opened and closed accrual.

2. Number of participants randomised.

3. Informed consent procedures.

4. Methods of random allocation.

5. Stratification factors (if any).

6. Methods of allocation concealment.

7. Blinding procedures for outcome assessment.

8. Details of the planned intervention in the drug arm: frequency, timing, doses.

9. Details of the planned intervention in the placebo arm: frequency, timing, doses.

#### Participant-level information – maternal characteristics at study entry

1. Unique identification coded for anonymity.

2. Maternal age.

3. Body mass index.

4. Parity.

5. Abdominal circumference.

6. Ethnicity.

7. Hypertensive disease (type of).

8. Uterine artery notching/pulsatility index.

9. Placental growth factor levels in maternal serum.

10. Treatment (e.g., aspirin, low molecular weight heparin, oral steroids, insulin).

#### Participant-level information – fetal characteristics at study entry

1. Estimated due date.

2. Date and time of randomisation.

3. Estimated fetal weight, abdominal circumference.

4. Absent or reversed end-diastolic flow of the umbilical artery, pulsatility index.

5. Pulsatility index of middle cerebral artery.

#### Participant-level information – maternal information after trial entry

1. Date and time of prenatal corticosteroid treatment.

2. Hypertensive disease (type of).

3. Highest blood pressure measured.

4. Proteinuria (maximum level recorded).

5. Platelet count (lowest recorded).

6. Adverse events and adverse effects for the woman at time of treatment and post-partum.

#### Participant-level information – fetal information after trial entry

1. Sequential assessments of the umbilical artery: absent or reversed end-diastolic flow, pulsatility index.

2. Sequential assessments of estimated fetal weight, abdominal circumference.

3. Sequential assessments of the middle cerebral artery: pulsatility index.

4. Estimated date and time if fetal death.

5. Adverse events and adverse effects for the fetus.

#### Participant-level information – neonatal information after trial entry

1. Unique baby information and mother identification coded for anonymity.

2. Date and time of delivery.

3. Birth weight, 5 minute Apgar scores, sex.

4. Mode of delivery.

5. Neonatal morbidity.

6. Date and time if neonatal death.

7. Childhood follow-up assessment.

8. Adverse events and adverse effects for the neonate.

#### Participant-level information – data on actual trial intervention

1. Type of treatment given (placebo, sildenafil, dose).

2. Number of doses actually given.

3. Interval between first dose and 32 weeks or delivery or fetal death.

4. Total actual drug exposure.

### Bias risk assessments

This project will use the Cochrane Collaboration methods for assessing risk of bias within individual trials [26]:

Allocation sequence generation

Low risk of bias: sequence generation was achieved using computer random number generation or a random number table. Drawing lots, tossing a coin, shuffling cards, and throwing dice are adequate if performed by an independent person not otherwise involved in the trial.

Uncertain risk of bias: the method of sequence generation was not specified.

High risk of bias: the sequence generation method was not random.

Allocation concealment

Low risk of bias: the participant allocations could not have been foreseen in advance of, or during, enrolment. Allocation was controlled by a central and independent randomisation unit. The allocation sequence was unknown to the investigators (for example, if the allocation sequence was hidden in sequentially numbered, opaque, and sealed envelopes).

Uncertain risk of bias: the method used to conceal the allocation was not described so that intervention allocations may have been foreseen in advance of, or during, enrolment.

High risk of bias: the allocation sequence was likely to be known to the investigators who assigned the participants.

Blinding of participants, personnel, and outcome assessors

Low risk of bias: blinding was performed adequately or the assessment of outcomes was not likely to be influenced by lack of blinding.

Uncertain risk of bias: there was insufficient information to assess whether blinding was likely to induce bias on the results.

High risk of bias: no blinding or incomplete blinding, and the assessment of outcomes were likely to be influenced by lack of blinding.

Incomplete outcome data

Low risk of bias: missing data were unlikely to make treatment effects depart from plausible values. Sufficient methods, such as multiple imputation, have been employed to handle missing data.

Uncertain risk of bias: there was insufficient information to assess whether missing data in combination with the method used to handle missing data were likely to induce bias on the results.

High risk of bias: the results were likely to be biased due to missing data.

Selective outcome reporting

Low risk of bias: all outcomes were pre-defined and reported or all clinically relevant and reasonably expected outcomes were reported.

Uncertain risk of bias: it was unclear whether all pre-defined and clinically relevant and reasonably expected outcomes were reported.

High risk of bias: one or more clinically relevant and reasonably expected outcomes were not reported, and data on these outcomes were likely to have been recorded.

For a trial to be assessed with low risk of bias in the selective outcome reporting domain, the trial should have been registered either on the http://www.clinicaltrials.gov website or a similar register, or there should be a protocol, e.g., published in a paper journal. In the case when the trial was run and published in the years when trial registration was not required, we carefully scrutinised all publications reporting on the trial to identify the trial objectives and outcomes. If usable data on all outcomes specified in the trial objectives were provided in the publications results section, then the trial was considered low risk of bias trial in the *Selective outcome reporting* domain.

Industry bias

Low risk of bias: the trial appears to be free of industry sponsorship that may manipulate the trial design, conductance, or results of the trial.

Uncertain risk of bias: the trial may or may not be free of for-profit bias as no information on clinical trial support or sponsorship is provided.

High risk of bias: the trial is sponsored by the industry.

Other bias

Low risk of bias: the trial appears to be free of other components that could put it at risk of bias.

Uncertain risk of bias: the trial may or may not be free of other components that could put it at risk of bias.

High risk of bias: there are other factors in the trial that could put it at risk of bias.

Trials assessed as having ‘low risk of bias’ in all of the individual domains specified in the review were considered ‘trials with low risk of bias’. Trials assessed as having ‘uncertain risk of bias’ or ‘high risk of bias’ in one or more of the specified in the review individual domains were considered trials with ‘high risk of bias’ [26-30].

### Dealing with missing data

We intend to perform an intention-to-treat analysis whenever possible. We will impute data for binary outcomes using various scenarios, namely best-best analysis, worst-worst analysis, best-worst analysis, and worst-best analysis.

For continuous outcomes, we will use available-participant analysis. We will impute the standard deviation from *P* values according to the instructions given in the *Cochrane Handbook for Systematic Reviews of Intervention*[26] and use the median for the meta-analysis when the mean is not available. If it is not possible to calculate the standard deviation from the *P* value or the confidence intervals (CIs), we will impute the standard deviation as the highest standard deviation in the other trials included under that outcome, fully recognising that this form of imputation will decrease the weight of the trial for calculation of mean differences and bias the effect estimate to no effect in the case of standardised mean difference.

### Outcomes

The primary outcome is defined as:

Neonatal survival until term age without serious neonatal morbidity defined as secondary outcomes 3–6. The outcome needs to be assessed formally at term age by clinic visit (or as a last-resort alternative by telephone).

Secondary outcomes are:

1. Any perinatal deaths (any know death irrespective of cause).

2. Perinatal deaths in normally formed infants (absence of severe congenital malformation, infection, or genetic abnormality).

3. Severe central nervous system injury (diagnosed by ultrasound and/or magnetic resonance imaging) periventricular leukomalacia grade II or more, or intracerebral haemorrhage grade III or more, or hydrocephalus.

4. Bronchopulmonary dysplasia (the requirement of ambulatory oxygen therapy >36 weeks corrected gestational age).

5. Retinopathy of prematurity (requiring treatment such as laser therapy; grade 2/3 or more).

6. Necrotising enterocolitis (requiring surgery).

7. Need for inotropes or vasopressors: number of days on inotropes or vasopressors, days on one, two, or three different inotropes or vasopressors.

8. Patent ductus arteriosus needing medical or surgical treatment.

9. Number of septic episodes, defined as culture proven or clinical, with need for antibiotics for 5 or more days.

10. Total number of days on artificial ventilation.

11. Postmenstrual age at discharge home.

12. Corrected age-adequate performance (both composite cognitive score [Mental Developmental Index] and composite motor score [Psychomotor Developmental Index] continuously and <85) on the two-year Bayley scales of infant and toddler development-III; cerebral palsy rate including severity scaling with Gross Motor Function Classification Scale and a Child Behaviour Check List -questionnaire.

13. Fetal growth velocity, measured by expected/observed average daily increase in ultrasound-estimated abdominal circumference.

14. Birth weight (grams, centiles).

15. Gestational age at delivery (days).

16. Co-incidence and severity of the maternal syndrome of pre-eclampsia/hemolysis, elevated liver enzymes, low platelets syndrome.

17. Maternal hypertension requiring treatment.

Safety monitoring (mother)

18. Serious adverse events.

19. Suspected unexpected serious adverse reaction.

20. Anything else considered an adverse event.

Safety monitoring (fetus)

21. Serious adverse events.

22. Suspected unexpected serious adverse reaction.

23. Anything else considered an adverse event.

### Statistical analyses

The detailed statistical analysis plan will be prepared and agreed upon by the STRIDER IPD Study Group prior to the data analyses. Any analyses conducted will be based on the checked and updated individual participant data from all available trials. All randomised participants with outcome data available will be included in the analyses, which will be performed on an intention-to-treat basis, according to the treatment allocation at randomisation.

For each of the outcomes (as well as the individual components of each composite outcome), a one-stage approach to analysis will be taken so that the IPD from all eligible trials are included in a single model. Fitting a single model for each outcome variable will enable the variation across trials to be accounted for within the model by including a fixed trial effect. A treatment by trial interaction term will be tested to assess heterogeneity of treatment effect across trials. If excessive statistical heterogeneity in treatment effect or inconsistency across trials is detected (that is, if the trial by treatment interaction term is significant), then the rationale for combining trials will be questioned and the source of heterogeneity explored.

Binary outcomes will be analysed using log binomial regression models and results will be presented as risk ratios with 95% CI and associated two-sided *P* values. Continuous outcomes will be analysed using linear regression models and results will be presented as differences in means with 95% CI and two-sided *P* values. Any differences in treatment effect between pre-specified subgroups of clinical characteristics at inclusion or at any time (gestational age, absent-reversed end-diastolic flow in the umbilical artery, placental growth factor maternal serum levels, maternal disease present or absent) will be assessed by testing a treatment by subgroup interaction term within the model.

### Trial sequential analysis

Trial sequential analysis will be applied to the meta-analyses combining IPD and aggregate data because cumulative meta-analyses are at risk of producing random errors due to sparse data and repetitive testing of the accumulating data [16-21]. The underlying assumption of trial sequential analysis is that testing for significance may be performed each time a new trial is added to the meta-analysis resulting in an increased risk of random errors. We will add the trials according to the year of publication, and if more than one trial is published in a year, the trials will be added alphabetically according to the last name of the first author. On the basis of the diversity-adjusted required information size, trial sequential monitoring boundaries will be constructed. These boundaries determine the statistical inference one may draw regarding the cumulative meta-analysis that has not reached the required information size; if the trial sequential monitoring boundary is crossed before the required information size is reached, firm evidence may perhaps be established and further trials may turn out to be superfluous. On the other hand, if the boundary is not surpassed, it will most probably be necessary to continue conducting trials in order to detect or reject a certain intervention effect.

We will apply trial sequential analysis using a diversity-adjusted required information size calculated from an alpha error of 0.05, a beta error of 0.20, the control event proportion obtained from the results of the meta-analysis, and a relative risk reduction of 20% for binary outcomes with two or more trials to determine whether more trials are necessary on this topic (if the trial sequential alpha-spending monitoring boundary or the futility zone is crossed, then more trials may be unnecessary). For continuous outcomes, the required sample size will be calculated from an alpha error of 0.05, a beta error of 0.20, the variance estimated from the meta-analysis results, and a minimal clinically relevant difference will be defined for each of the continuous outcomes.

### Planned subgroup analyses

We plan the following subgroup analyses:

● IPD trials compared to trials only providing aggregate data.

● Trials with low risk of bias compared to trials with high risk of bias.

● Trials with dose of intervention below the median compared to trials with dose of intervention above the median.

### Planned sensitivity analyses

To assess whether the results are robust to trial design and quality, sensitivity analyses will be performed on the primary outcome to test changes of the results after exclusion of trials with smaller sample size, centres with smaller sample size, and trials/centres with high rate of missing values. We will impute data for binary missing outcomes using various scenarios, namely best-best analysis, worst-worst analysis, best-worst analysis, and worst-best analysis.

### Multiple comparisons

A very large number of outcomes are being investigated in this study, which increases the risk of observing ‘false positive’ results due to multiplicity. However, all outcomes are important in giving a full clinical picture that considers the benefits and risks to both mothers and infants. We do not plan formal statistical adjustment of *P* values to account for multiple comparisons due to the non-independence of outcomes in this study. Results will be interpreted with caution. Decisions as to whether the intervention should be recommended will be based primarily on the primary outcome of the fetus and will consider any adverse effects on the mother and/or child.

### Ethics and management issues

Participants in the individual trials have given informed consent to participate in their respective trial. The data for this project are to be used for the purpose for which they were originally collected and are available through an agreement between all trialists of the collaborative group. These trialists remain the custodians of their original individual trial data at all times.

### Project management

For the purpose of this project, the international STRIDER IPD Study Group will manage the project. Additionally, the coordinating investigator of each trial will be invited to become a member of the STRIDER IPD Study Group. The Study Group and statisticians from the data management centre will develop the statistical analysis plan. The data management centre will be responsible for the storage and analyses of the IPD project data.

### The Study Group meetings

Study Group face-to-face meetings will be organised at least twice during the study. Representatives of all eligible trials will be invited to attend those meetings. The meetings will be scheduled, if possible, in conjunction with international conferences. During those meetings, various aspects of the project will be discussed with all the collaborators, such as the project design and conduct, the analysis plan, and the interpretation and reporting of the results.

The final results of the study will be presented to the collaborators for discussion. The main manuscript will be prepared by the STRIDER IPD Study Group. The revised draft paper will be circulated for final comment and agreement prior to publication. Publications arising from these data will be authored by all members of the STRIDER IPD Study Group on behalf of all other collaborators participating, who will be acknowledged within the manuscript.

## Discussion

Severe early-onset FGR due to placental insufficiency is associated with a high risk of perinatal morbidity with long-lasting sequelae and mortality. Placental insufficiency is the result of abnormal formation and function of the placenta (placentation) with inadequate remodelling of the maternal spiral (uteroplacental) arteries. There is currently no therapy available with demonstrated effectiveness, making monitoring and timely delivery of the child the only treatment option. Both *in vitro* and *in vivo* evidence suggests sildenafil citrate as a therapeutic strategy to improve uteroplacental blood flow, fetal growth, and meaningful outcomes in FGR pregnancies.

On the basis of preliminary research, some centres are already adopting the treatment with sildenafil. There is, however, significant uncertainty regarding the true health benefits, if any. Moreover, potential harm is not yet excluded. Fetotoxicity has not been described yet, but is theoretically possible. For these reasons, evidence from randomised clinical trials is needed before widespread implementation [31].

Given the sample size issues, no single randomised clinical trial will ultimately be likely to give the answers to the questions posed in this study. For this reason, simultaneous smaller and medium-sized randomised clinical trials have been planned and funded. The present pre-planned systematic review with IPD meta-analysis will allow more definite answers, including effect modifications in relevant subgroups. Furthermore, the totality of evidence from IPD trials and trials reporting only aggregate data can be assessed and evaluated with GRADE methodology [32]. All weighing of results should take into consideration risks of bias and aim to minimise these: systematic errors (bias) due to methodology and design; risks of academic bias if there are conflicting results between studies from this GONet group and other studies; risks of random errors (play of chance) [33]. According to our knowledge, this is the first time a protocol for a systematic review has been finalised and published before the first larger trial has been launched on a topic. With the AllTrials initiative and other initiatives for transparency in clinical research, we hope that the number of trials providing IPD for this systematic review will be large.

## Abbreviations

FGR: Fetal growth restriction; GONet: Global obstetrics network; IPD: Individual participant data; NICU: Neonatal intensive care unit; STRIDER: Sildenafil therapy in dismal prognosis early-onset intrauterine growth restriction; UBC: University of British Columbia.

## Competing interests

None of the authors have competing interests to report. Authors PB, ZA, LK, AP, WG, BM, AvW, and PvD are involved in successful/ongoing funding applications for the studies that form the basis of this IPD (Health Research Council, New Zealand; UK Medical Research Council, United Kingdom; ZonMW, The Netherlands; and CIHR, Canada).

## Authors’ contributions

WG participated in the study design and drafted the manuscript. ZA participated in the design of the study and reviewed the manuscript. PvD developed the hypotheses, participated in the design of the study, instigated the international collaborations, and reviewed the manuscript. LK developed the hypotheses, participated in the design of the study, instigated the international collaborations, and reviewed the manuscript. AP participated in the design of the study and reviewed the manuscript. AvW participated in the design of the study particularly neonatal outcome analysis and reviewed the manuscript. CG participated in the design of the study, particularly the statistics section and reviewed the manuscript. BM participated in the design of the study and reviewed the manuscript. PB developed the hypotheses, participated in the design of the study, instigated the international collaborations, and reviewed the manuscript. All authors read and approved the final manuscript.
